# Predictive model for severe COVID-19 using SARS-CoV-2 whole-genome sequencing and electronic health record data, March 2020-May 2021

**DOI:** 10.1371/journal.pone.0271381

**Published:** 2022-07-12

**Authors:** Lei Zhu, Jane W. Marsh, Marissa P. Griffith, Kevin Collins, Vatsala Srinivasa, Kady Waggle, Daria Van Tyne, Graham M. Snyder, Tung Phan, Alan Wells, Oscar C. Marroquin, Lee H. Harrison

**Affiliations:** 1 Microbial Genomic Epidemiology Laboratory, Center for Genomic Epidemiology, University of Pittsburgh, Pittsburgh, Pennsylvania, United States of America; 2 Division of Infectious Diseases, University of Pittsburgh School of Medicine, Pittsburgh, Pennsylvania, United States of America; 3 Healthcare Data and Analytics, University of Pittsburgh Medical Center, Pittsburgh, Pennsylvania, United States of America; 4 Department of Infection Prevention and Control, University of Pittsburgh Medical Center, Pittsburgh, Pennsylvania, United States of America; 5 Department of Pathology, University of Pittsburgh, Pittsburgh, Pennsylvania, United States of America; Universita degli Studi di Roma Tor Vergata, ITALY

## Abstract

**Objective:**

We used SARS-CoV-2 whole-genome sequencing (WGS) and electronic health record (EHR) data to investigate the associations between viral genomes and clinical characteristics and severe outcomes among hospitalized COVID-19 patients.

**Methods:**

We conducted a case-control study of severe COVID-19 infection among patients hospitalized at a large academic referral hospital between March 2020 and May 2021. SARS-CoV-2 WGS was performed, and demographic and clinical characteristics were obtained from the EHR. Severe COVID-19 (case patients) was defined as having one or more of the following: requirement for supplemental oxygen, mechanical ventilation, or death during hospital admission. Controls were hospitalized patients diagnosed with COVID-19 who did not meet the criteria for severe infection. We constructed predictive models incorporating clinical and demographic variables as well as WGS data including lineage, clade, and SARS-CoV-2 SNP/GWAS data for severe COVID-19 using multiple logistic regression.

**Results:**

Of 1,802 hospitalized SARS-CoV-2-positive patients, we performed WGS on samples collected from 590 patients, of whom 396 were case patients and 194 were controls. Age (p = 0.001), BMI (p = 0.032), test positive time period (p = 0.001), Charlson comorbidity index (p = 0.001), history of chronic heart failure (p = 0.003), atrial fibrillation (p = 0.002), or diabetes (p = 0.007) were significantly associated with case-control status. SARS-CoV-2 WGS data did not appreciably change the results of the above risk factor analysis, though infection with clade 20A was associated with a higher risk of severe disease, after adjusting for confounder variables (p = 0.024, OR = 3.25; 95%CI: 1.31–8.06).

**Conclusions:**

Among people hospitalized with COVID-19, older age, higher BMI, earlier test positive period, history of chronic heart failure, atrial fibrillation, or diabetes, and infection with clade 20A SARS-CoV-2 strains can predict severe COVID-19.

## Introduction

COVID-19 is a respiratory illness caused by Severe Acute Respiratory Syndrome Coronavirus 2 (SARS-CoV-2) and was initially reported in Wuhan, China in December 30, 2019 [1, 2]. As of March 2022, the COVID-19 pandemic has caused infection of more than 437 million individuals and more than 5.9 million deaths globally, [3] including more than 79 million cases and over 955,000 deaths in the U.S. [3]. The social and economic impacts of this disease are enormous and it is crucial to understand the factors that affect the risk of severe COVID-19 so that limited hospital resources can be prioritized and intervention strategies can be made accordingly.

Epidemiologic studies have investigated the risk factors of severe COVID-19. Most studies used electronic health record (EHR) data for risk factor evaluation [4–6]. Age, gender, C-reactive protein level, and comorbidities are commonly reported as risk factors for severe COVID-19 [4–6]. Specific conditions listed by Centers for Disease Control and Prevention (CDC) as being associated with severe COVID-19 include chronic kidney disease, chronic lung disease (e.g., chronic obstructive pulmonary disease (COPD), asthma, cystic fibrosis), diabetes, heart conditions (e.g., congestive heart failure, coronary artery disease, hypertension), obesity, solid organ transplant, and sickle cell disease [7].

A small number of genomic epidemiologic studies have explored the association of host and pathogen genetic variation and COVID-19 severity [8–11]. A study by Dite G et al., using UK biobank data, performed a GWAS in the human genome to identify single nucleotide polymorphisms (SNPs) associated with disease severity using a SNP score, as well as the impact of demographic and comorbidity risk factors on severe COVID-19 severity [12]. Those findings showed that the effect of age, comorbidities, and/or gender plus viral genetic factors predicted the risk of severe COVID-19 more accurately than demographic and comorbidity risk factors alone. In the Dite et al. study, a model including age, gender, comorbidities, and human SNP score discriminated severe COVID-19 better than clinical factors alone, or SNP score alone [12].

In the present study, we utilized EHR and SARS-CoV-2 whole-genome sequencing (WGS) data among patients from the University of Pittsburgh Medical Center-Presbyterian Hospital (UPMC), to assess the association between potential risk factors and COVID-19 clinical outcomes. The main study aim was to investigate the association between viral genomic and clinical characteristics to build models that can accurately predict risk of severe COVID-19.

## Materials and methods

### Setting and study design

This was a case-control study that was conducted at UPMC, a large academic referral hospital. Residual SARS-CoV-2 polymerase chain reaction (PCR)-positive samples were collected from hospitalized UPMC patients. All samples were derived from residual nasopharyngeal swab specimens, after the performance of all clinical testing for diagnosis of COVID-19 in the UPMC Clinical Laboratories. Ethics approval was obtained from the University of Pittsburgh institutional review board.

### Clinical and demographic data extraction from electronic health records

Health records of SARS-CoV-2 positive patients were accessed through the UPMC EHR system. Comorbidities that have consistently been found to be associated with an increased risk of severe COVID-19 and those that might increase the risk were extracted [7]. Comorbidities were categorized as present or not present. Additionally, Charlson Comorbidity Index (CCI) was calculated for each patient [13].

### Case-control study of severe COVID-19 and associated risk factors

Severe COVID-19 (case patients) was defined as a hospitalized SARS-CoV-2-positive patient (diagnosed by RT-PCR) with at least one of the following severe outcomes: treatment with supplemental oxygen, mechanical ventilation, both within 30 days before and after the positive SAR-CoV-2 test, or in-hospital mortality. Control patients were those who were hospitalized and positive for SARS-CoV-2, but without any of the above severe outcomes. For patients who tested positive for SARS-CoV-2 multiple times during the study period, only the first positive sample was included unless the interval between positive tests exceeded 90 days, in which case infections were counted as separate episodes. Body mass index (BMI) was grouped into normal, overweight, and obese groups using the cut-offs <25, 25–30, and ≥30 kg/m^2^, respectively. We grouped race as “others” for Asian, American Indian, not specified, or declined to respond, to compare those with Caucasian and African American groups. Due to the small sample size, we also included Hispanic, not specified, and declined as one group, to compare to non- Hispanic or Latino patients. In our study, race was missing for 214 patients, ethnicity was missing for 215 patients, BMI was missing for 36 patients, and CCI was missing for 88 patients. We classified case patients and controls according to the time period during which they were tested: 1) March (when SARS-CoV-2 first appeared in our region)-June 2020, 2) July-October 2020, 3) November 2020- February 2021, and 4) March-May 2021.

Patients were grouped into COVID-19 severe outcome (case patients) vs. mild infection (controls). Frequency matching by time period was used to select controls ensuring that cases and controls had the same distribution over the test positive period. Using this design, controls were randomly selected to match with cases; we aimed to include one case for each control. WGS was conducted for all selected cases and controls, and only those samples that passed laboratory quality control measures (below) were included in the phylogenetic analyses. Potential covariates included age, gender, race, ethnicity (White vs. African American vs. any other ethnicity), body-mass index (BMI), and comorbidities.

### Sample selection, inclusion, and exclusion for the study cohort

**Fig 1:**
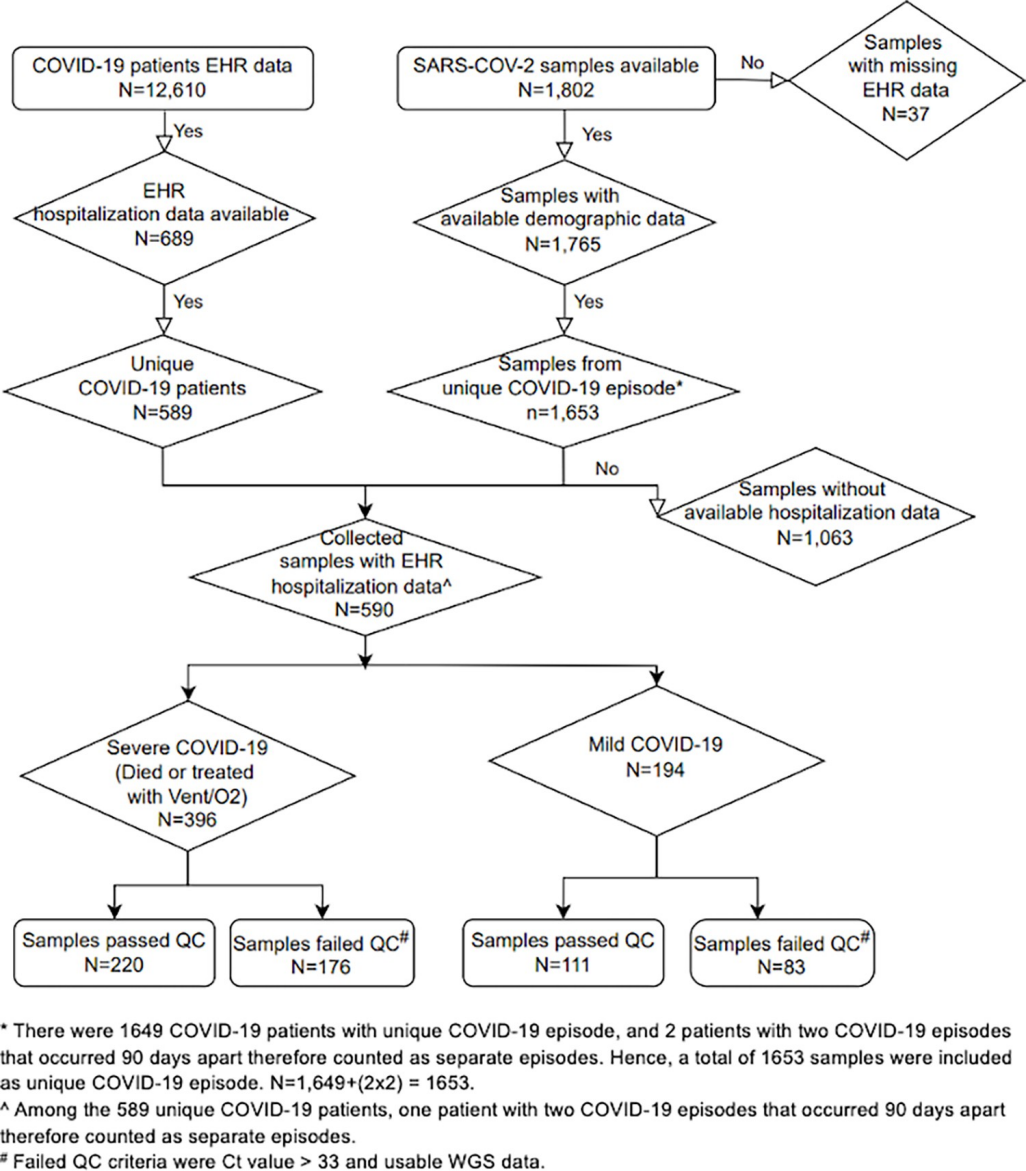
Sample selection, inclusion, and exclusion of the analysis cohort, UPMC, March 2020- May 2021.

We utilized stored SARS-CoV-2 genomic data (N = 1,802) and UPMC collected hospitalization, demographic, and clinical data (N = 12,610) to generate our analysis cohort for COVID-19 patients. Patient MRN or hospital account number were used to merge the databases. COVID-19 testing dates were used to verify COVID-19 episode-related treatments for the included patients. More detailed inclusion and exclusion criteria and the data reduction processes used to form the study cohort are included in Fig 1. All UPMC patients with SARS-CoV-2 positive tests from March 2020-May 2021 and hospitalization data from the EHR were eligible to be included in the study. We then further determined patient COVID-19 case-control status based on the above criteria.

### Clinical sample collection and WGS

Residual SARS-CoV-2-positive samples were collected from the UPMC clinical microbiology laboratory from March 2020 to May 2021. Total RNA was extracted using the QiaAmp Viral RNA Mini kit (Qiagen) according to the manufacturer’s instructions. SARS-CoV-2 viral load was determined using the CDC diagnostic N1 primer/probe set and the TaqPath^TM^ 1-Step RT-qPCR kit (ThermoFisher Scientific). Samples with cycle threshold (Ct) less than 33 were subjected to WGS (263 of the 396 cases and 130 of the 194 controls at the time of the initial diagnostic nasopharyngeal swab) using either the ARTICv3 or Illumina RNA enrichment with the respiratory virus oligonucleotide panel v2 according to manufacturer’s protocol [14]. Libraries were sequenced on a NextSeq 550 high output flow cell. Reads were mapped to the Wuhan-1 reference genome (NC_045512.2) using Breseq v0.33.2. Genomes with >40X average coverage and less than 5% ambiguous nucleotides were included in the phylogenetic analysis; samples with genomes not meeting those criteria were classified as failed WGS. Multiple sequence alignments to the reference genome (NC_045512.2) was performed using MAFFT v7.475 and maximum likelihood trees were generated with RAxML v8.2.12 using the general time reversible model of evolution (GTRCAT). The resulting alignment file was used to obtain a time-based phylogenetic tree using treetime v0.8.1. The phylogenetic tree was visualized using ggtree v3.2.1 package in R v4.1.1.

### Genome-wide association study (GWAS)

A total of 1,202 SNPs from 331 SARS-CoV-2 genomes were studied. Chi-square and Fisher’s exact tests were performed to compare SNP differences between cases and controls. To identify potential viral genetic variations associated with the COVID-19 severity, we also performed a GWAS using TreeWAS v1.0, a phylogenetic-based approach for GWAS of microbial genomes that considers the observed virus population structure [15] using the maximum likelihood tree generated by RAxML. To determine statistical significance of the difference in mutations between cases and controls, GWAS summary (Manhattan) plots of the association statistics were generated, adjusting for multiple comparisons using the Bonferroni correction.

### Statistical analysis

Univariate analyses were conducted for both categorical and continuous variables. All continuous variables were evaluated for normality. Mean and standard deviation (SD) were presented for normally distributed variables. Median and interquartile range (IQR) were presented for variables that were not normally distributed. Percentage and total number were presented for categorical variables. Bivariate analyses were conducted using Chi-square or Fisher’s exact test for categorical variables and outcomes. Two samples T-tests or Wilcoxon-Mann-Whitney tests were used for continuous variables and outcomes.

Multivariable regression analyses were conducted to determine which demographic variables, clinical risk factors, or genomic profiles (e.g., lineage, SNPs) were associated with severe COVID-19. These potential covariates (e.g., age, gender, BMI) and selected covariates (based on the bivariate analyses’ results) were included in the logistic regression model to assess associations with the outcome of severe versus mild COVID-19. As BMI is correlated with obesity, we selected one member of each pair to include in the model. Model selections were conducted based on the Akaike information criterion [16]. Hosmer and Lemeshow goodness of fit tests were performed. We further evaluated the performance of our classification model using ROC analysis and calculated area under the curve (AUC) values. Higher AUC indicates better model performance of distinguishing between the cases and controls, therefore, models with higher AUC were judged as better at predicting severe outcome.

All statistical analyses were performed using SAS software version 9.4 (SAS Institute Inc., Cary, NC, USA) and R Statistical Software (Version 4.0.2). Two-sided p-values <0.05 were considered statistically significant.

## Results

Of 1,802 hospitalized SARS-CoV-2-positive patients identified between March 2020 and May 2021, we performed WGS analysis on 590 samples (Fig 1). Of the patients with available demographic and clinical data, 287 patients were women (49%), 303 were men (51%), 251 were White (43%), 114 were African American (19%), and 351 were non-Hispanic or Latino (60%). A total of 277 patients were obese (47%), 145 were overweight (25%), and 132 had normal BMI (22%). Selected patients had median BMI of 30 (Interquartile range (IQR): 25.9–35.9); and mean age on test positive date of 64 (standard deviation (SD) = 18.0). The range of the CCI was 0–11 (mean ± SD: 2 ± 1.85). Twenty-three percent of samples were from March-June 2020, 22% were from July-October 2020, 36% were from November 2020- February 2021, and 19% were from March-May 2021 (Table 1).

**Table 1:** Demographic characteristics and treatment and disease history information of UPMC COVID-19 inpatients.

	Total N, %	Severe COVID-19 N, %	Mild COVID-19 N, %	p value*
	590, 100%	396, 67%	194, 33%	
Age (Categorical)				
<30	31, 5%	11, 3%	20, 10%	
30–49	77, 13%	42, 11%	35, 18%	
50–69	245, 42%	176, 44%	69, 36%	
70+	237, 40%	167, 42%	70, 36%	< .0001
Gender				
Male	303, 51%	205, 52%	98, 51%	
Female	287, 49%	191, 48%	96, 49%	0.775
BMI				
Normal (<25kg/m^2^)	132, 22%	81, 20%	51, 26%	
Overweight (25-<30kg/m^2^)	145, 25%	90, 23%	55, 28%	
Obese (≥30kg/m^2^)	277, 47%	200, 51%	77, 40%	
Not Available	36, 6%	25, 6%	11, 6%	0.032
Race				
White	251, 43%	164, 41%	87, 45%	
Black	114, 19%	71, 18%	43, 22%	
Others	11, 2%	6, 2%	5, 3%	
Not Available	214, 36%	155, 39%	59, 30%	0.681
Ethnicity				
Hispanic and Others	24, 4%	11, 3%	13, 7%	
Non-Hispanic or Latino	351, 60%	229, 58%	122, 63%	
Not Available	215, 36%	156, 39%	59, 30%	0.055
Test Positive Period				
March-June, 2020	134, 23%	109, 28%	25, 13%	
July-October, 2020	129, 22%	85, 21%	44, 23%	
November, 2020- February, 2021	214, 36%	130, 33%	84, 43%	
March-May, 2021	113, 19%	72, 18%	41, 21%	0.001
Charlson Comorbidity Index (CCI)				
0	184, 31%	109, 28%	75, 39%	
1	122, 21%	82, 21%	40, 21%	
2	73, 12%	47, 12%	26, 13%	
3	47, 8%	31, 8%	16, 8%	
4	29, 5%	21, 5%	8, 4%	
5+	47, 8%	38, 10%	9, 5%	
Not Available	88, 15%	68, 17%	20, 10%	0.114
History of Chronic Heart Failure	90, 15%	71, 18%	19, 10%	0.003
History of Cancer	80, 14%	51, 13%	29, 15%	0.745
History of Atrial fibrillation	59, 10%	49, 12%	10, 5%	0.002
History of Asthma	159, 27%	107, 27%	52, 27%	0.531
History of Coronary Artery Disease	91, 15%	67, 17%	24, 12%	0.066
History of Cystic Fibrosis	1, 0%	1, 0%	0, 0%	1^#^
History of COPD	283, 48%	176, 44%	107, 55%	0.166
History of Diabetes	169, 29%	124, 31%	45, 23%	0.007
History of Hypertension	282, 48%	190, 48%	92, 47%	0.278
History of Inflammatory Bowel Disease	10, 2%	6, 2%	4, 2%	0.744^#^
History of Obesity	321, 54%	219, 55%	102, 53%	0.070
History of Psoriatic Arthritis	10, 2%	7, 2%	3, 2%	1^#^
History of Rheumatoid Arthritis	18, 3%	10, 3%	8, 4%	0.374
History of Chronic Kidney Disease	70, 12%	50, 13%	20, 10%	0.248
History of Solid Organ Transplant	35, 6%	20, 5%	15, 8%	0.190
History of Sickle Cell	5, 1%	3, 1%	2, 1%	0.665^#^
Monoclonal Antibody Infusion	11, 2%	8, 2%	3, 2%	0.689
History of Antiplatelet Meds	164, 28%	113, 29%	51, 26%	0.243
Bamlanivimab use only	5, 1%	5, 1%	0, 0%	0.178^#^
Casirivimab and Imdevimab use	6, 1%	3, 1%	3, 2%	0.400^#^
Dexamethasone use	233, 39%	197, 50%	36, 19%	< .0001
Remdesivir use	208, 35%	178, 45%	30, 15%	< .0001
Bilevel positive airway pressure (BiPap) use	98, 17%	90, 23%	8, 4%	< .0001
Hydroxycholoroquine use	48, 8%	45, 11%	3, 2%	< .0001^#^
Tocilizumab use	15, 3%	14, 4%	1, 1%	0.003^#^
Ventilation				
No	477, 81%	283, 71%	194, 100%	
Yes	113, 19%	113, 29%	0, 0%	<0.001
Oxygen therapy				
No	206, 35%	12, 3%	194, 100%	
Yes	384, 65%	384, 97%	0, 0%	<0.001
Death				
No	506, 86%	312, 79%	194, 100%	
Yes	84, 14%	84, 21%	0, 0%	<0.001

*Chi-squared or Fisher’s exact tests’ p-values were generated for categorical variables; and t tests or Wilcoxon-Mann-Whitney test p-values were generated for continuous variables

#Fisher’s exact test p-value

^Wilcoxon-Mann-Whitney test p-value

Total sample size is 590. Since our data were obtained from EHR, we expected missing data for those demographic and clinical variables. Among the above variables, race was missing for 214 patients; ethnicity was missing for 215patients; and BMI was missing for 36 patients. Charlson comorbidity index, history of chronic heart failure, cancer, atrial fibrillation, asthma, coronary artery disease, cystic fibrosis, pulmonary fibrosis, diabetes, hypertension, inflammatory bowel disease, obesity, psoriatic arthritis, rheumatoid arthritis, and chronic kidney disease and history of antiplatelet medication use have 88 missing data. History of COPD was missing for 215patients. History of solid organ and history of sickle cell were missing for 1 patient; and Dexamethasone use, Remdesivir use, BiPap use, Hydroxycholoroquine use, and Tocilizumab use were missing for 2 patients.

Among the 590 patients included in the analysis, 396 were treated with either ventilation (n = 113) or oxygen therapy (n = 384) and 84 died during hospitalization. Frequency-matched controls (N = 194) were randomly selected from March 2020 to May 2021 for WGS. Cases ranged in age from 3 to 101 years with a mean of 67 years (SD: 16.31), which was older than controls (mean ± SD: 60 ± 20.21, range: 4 to 96 years) (p = 0.001). Median body mass index (BMI) was 31 kg/m^2^ (IQR: 26.22–37.00) for cases and 28.4 kg/m^2^ (IQR: 24.60–34.40) for controls. Forty-eight percent of cases were women (vs. 52% of men) while 49% of controls were women (compared to 51% of men) (Table 1). The median CCI for cases was 1 (IQR: 0–3; mean ± SD: 2 ±1.42) and controls was 1 (IQR: 0–2; mean ± SD:1 ± 1.48). Comparing cases to controls, patients’ age, test positive period, mean CCI, history of heart failure, atrial fibrillation, and diabetes, were all statistically different (p<0.05). As expected, some COVID-19 treatments, including dexamethasone, remdesivir, bilevel positive airway pressure (BiPaP), hydroxychloroquine, and tocilizumab use were more common in case patients than controls (p<0.05). (Table 1).

**Fig 2:**
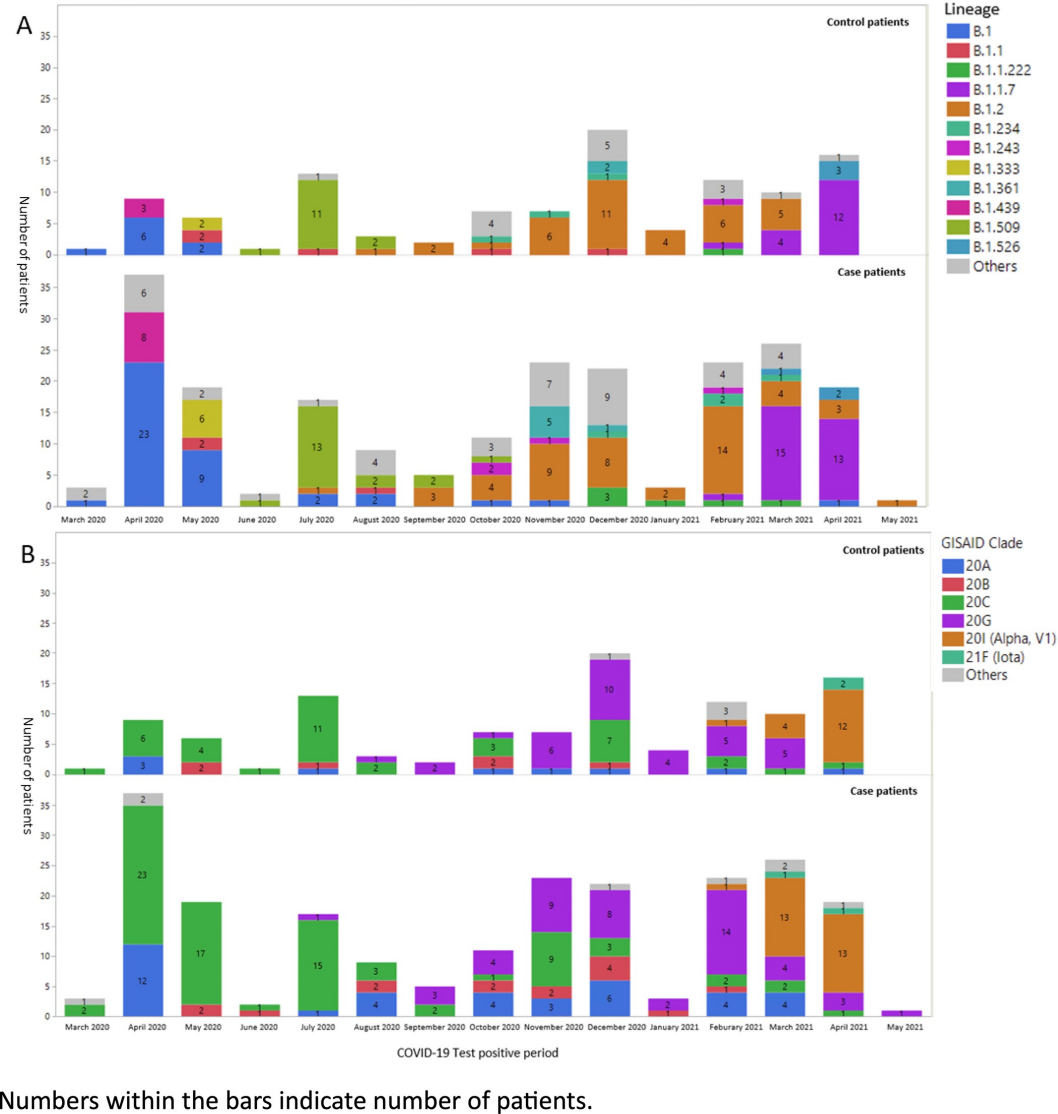
Emergence of SARS-CoV-2 lineages (Panel A) and clades (Panel B) over the study period (March 2020 to May 2021), comparing control vs. case patients (mild vs. severe COVID-19) at UPMC.

WGS data from 220 cases and 111 controls were included in the phylogenetic analyses (Fig 2, Table 2). Compared to controls, cases had a significantly higher percentage of B.1 and Clade 20a lineages, and a lower percentage of lineage B.1.2. Our genome-wide association study (GWAS) analyses found five mutations (ORF1b P1975S, S A622V, ORF3a G172V, ORF7a S83L, N P199L) that were associated with severe COVID-19, based on Chi-square or Fisher’s exact tests. In the Manhattan plots with the Bonferroni adjustment for multiple comparisons, however, these mutations were no longer statistically significant (Manhattan plot terminal/ simultaneous/ subsequent p values were all not significant). TreeWAS also failed to detect any significant associations between and SARS-CoV-2 mutations and case or control status, based on simultaneous, terminal, and subsequent scores. The time-based phylogenetic tree (S1 Fig) and descriptive bar plots (Fig 2) illustrate the emergence of lineages over time from B.1 (March-June 2020) and B.1.509 (July-October 2020) to B.1.2 (November 2020-Febuary 2021) and B.1.1.7 (March-May 2021), for both cases and controls. More specifically, in the spring of 2020, most SARS-CoV-2 lineages belonged to the B.1 (and some B.1.439) lineage. From summer (June) 2020 to early 2021, a mixture of lineages emerged, notably B.1.509 followed by B.1.2, with a few other lineages present at lower frequency (B.1.361). Samples from early spring of 2021 were dominated by B.1.1.7 (alpha) and a few other lineages (B.1.2 and B.1.526). (Fig 2).

**Table 2:** Summary of SARS-CoV-2 lineages, clades, and individual mutations (gene) of UPMC COVID-19 inpatients.

	Level	Total N, %	Severe COVID-19 N, %	Mild COVID-19 N, %	p-value*
	All	331, 100%	220, 66%	111, 34%	
Lineage	B.1	49, 15%	40, 18%	9, 8%	0.015
	B.1.1	8, 2%	3, 1%	5, 5%	0.124^#^
	B.1.1.222	7, 2%	6, 3%	1, 1%	0.431^#^
	B.1.1.7	46, 14%	29, 13%	17, 15%	0.596
	B.1.2	85, 26%	49, 22%	36, 32%	0.046
	B.1.234	7, 2%	4, 2%	3, 3%	0.691^#^
	B.1.243	5, 2%	4, 2%	1, 1%	0.667^#^
	B.1.333	8, 2%	6, 3%	2, 2%	0.723^#^
	B.1.361	8, 2%	6, 3%	2, 2%	0.723^#^
	B.1.439	11, 3%	8, 4%	3, 3%	0.757^#^
	B.1.509	33, 10%	19, 9%	14, 13%	0.254
	B.1.526	6, 2%	3, 1%	3, 3%	0.407^#^
	Others^	58, 18%	43, 20%	15, 14%	0.173
Clade	20A	47, 14%	38, 17%	9, 8%	0.024
	20B	21, 6%	15, 7%	6, 5%	0.619
	20C	120, 36%	81, 37%	39, 35%	0.764
	20G	83, 25%	49, 22%	34, 31%	0.098
	20I (Alpha, V1)	44, 13%	27, 12%	17, 15%	0.441
	21F (Lota)	4, 1%	2, 1%	2, 2%	0.604^#^
	Others	12, 4%	8, 4%	4, 4%	1.000
ORF1b: P1975S		16, 3%	7, 2%	9, 5%	0.049
S: A622V		5, 1%	1, 0%	4, 2%	0.046^#^
ORF3a: G172V		85, 14%	49, 12%	36, 19%	0.046
ORF7a: S83L		12, 2%	4, 1%	8, 4%	0.025^#^
N: P199L		89, 15%	51, 13%	38, 20%	0.032

*Chi-squared or Fisher’s exact tests’ p-values were generated for categorical variables. Those ones marked with # are Fisher’s exact test p-values

^Other lineages (with N<5) includes: B.1.400, B.1.582, B.1.1.207, B.1.110.3, B.1.240, B.1.306, B.1.311, B.1.332, B.1.349, B.1.541, B.1.596, B.1.605, C.23, A, A.17, B.1.1.1, B.1.1.225, B.1.1.304, B.1.1.316, B.1.1.337, B.1.1.447, B.1.351, B.1.369, B.1.396, B.1.422, B.1.427, B.1.429, B.1.448, B.1.507, B.1.540, B.1.556, B.1.565, and B.28.

For analysis of Ct values, we included all samples, regardless of whether or not they had usable WGS data (total N = 334 for cases and N = 44 for control). The median Ct value of the samples was 27.8 (IQR: 21.10–34.35 cycles). Cases had a slightly lower median Ct value compared with the control group (27.8 [IQR: 20.95–34.60] vs. 28.6 [IQR: 22.75–32.80]), suggesting that cases had higher viral titer than controls. Among samples that passed QC (defined as RT-qPCR mean Ct<33), cases (N = 201) had even lower median Ct value (22.1, IQR: 18.70–25.90) than controls (N = 28) (24.9, IQR: 19.65–28.00). This data suggests that patients in the case group had higher viral loads at the time they were sampled compared with patients in the control group.

The unadjusted and adjusted odds ratios for the included covariates are presented in Table 3. Age, BMI, test positive period, CCI, history of chronic heart failure, atrial fibrillation, or diabetes, presence of lineage B.1, or B.1.2, and Clade 20A were all independently associated with severe COVID-19. After adjusting for age, history of obesity was also significantly associated with severe COVID-19; though presence of lineage B.1.2 was no longer associated with the outcome. (Table 3).

**Table 3:** Predictive models for risk of severe COVID-19 among UPMC inpatients.

		Unadjusted model		Model Adjusted for Age		Model Adjusted for Selected Covariates		Model for patients with WGS data	
Variables	Level	Odds Ratio	95% Confidence Interval	Odds Ratio	95% Confidence Interval	Odds Ratio	95% Confidence Interval	Odds Ratio	95% Confidence Interval
Age	Per each year of increasing age	1.02	1.01–1.03	Adjusted		1.02	1.01–1.03	1.02	1.00–1.04
Male	Male vs. Female	1.05	0.75–1.48	1.01	0.71–1.43				
BMI	Per 1kg/m^2^ of increasing BMI	1.02	1.00–1.04	1.04	1.01–1.06	1.03	1.00–1.05	1.00	0.98–1.03
Race	Black vs. White	0.64	0.19–2.15	0.61	0.18–2.08				
	Others vs. White	0.88	0.55–1.39	1.02	0.63–1.63				
Hispanic and others	Hispanic and others vs. Non-Hispanic or Latino	0.45	0.20–1.04	0.51	0.22–1.20				
Test positive period*	July-October, 2020	0.44	0.25–0.78	0.46	0.26–0.83	0.40	0.21–0.76	0.45	0.20–1.02
	November 2020- February 2021	0.36	0.21–0.59	0.38	0.23–0.64	0.35	0.20–0.64	0.47	0.22–1.00
	March-May, 2021	0.40	0.23–0.72	0.45	0.25–0.80	0.32	0.17–0.62	0.46	0.20–1.05
CCI	Per each unit increasing CCI	1.18	1.06–1.32	1.14	1.01–1.27				
History of chronic heart failure		2.25	1.31–3.88	2.07	1.20–3.59	1.59	0.88–2.90	1.79	0.79–4.06
History of atrial fibrillation		2.88	1.42–5.84	2.35	1.14–4.82	2.08	0.91–4.75	2.99	0.94–9.50
History of coronary artery disease		1.60	0.97–2.67	1.29	0.76–2.18				
History of hypertension		1.23	0.85–1.78	1.04	0.71–1.53				
History of diabetes		1.74	1.16–2.62	1.64	1.09–2.47	1.53	0.98–2.39	1.91	1.07–3.41
History of obesity		1.42	0.97–2.07	1.61	1.09–2.39				
History of chronic kidney disease		1.39	0.80–2.41	1.30	0.74–2.28				
History of cancer		0.92	0.56–1.52	0.71	0.42–1.19				
History of asthma		1.14	0.76–1.69	1.18	0.79–1.78				
History of cystic fibrosis		>999.99	<0.001 - >999.99	>999.99	<0.001 - >999.99				
History of pulmonary fibrosis		>999.99	<0.001 - >999.99	>999.99	<0.001 - >999.99				
History of COPD		1.43	0.86–2.39	1.41	0.84–2.36				
History of inflammatory bowel disease		0.79	0.22–2.85	0.85	0.23–3.11				
History of psoriatic arthritis		1.24	0.32–4.87	1.17	0.29–4.69				
History of rheumatoid arthritis		0.65	0.25–1.69	0.61	0.23–1.58				
History of solid organ transplant		0.63	0.32–1.26	0.68	0.34–1.37				
History of sickle cell		0.73	0.12–4.40	1.35	0.22–8.50				
Ct value (mean)		1.00	0.95–1.04	1.00	0.96–1.04				
Lineage B.1		2.31	1.10–4.86	2.39	1.10–5.18				
Lineage B.1.2		0.62	0.39–0.99	0.65	0.39–1.10				
Clade 20A		2.18	1.03–4.61	2.45	1.12–5.34			3.25	1.31–8.06

* Reference group for test positive period is March-June, 2020

Several different candidate models were constructed, and their ROC curves were evaluated. The four models we selected to present were: 1) unadjusted model, 2) model adjusted for age; 3) model that includes final selected covariates, and 4) model that includes final selected covariates and WGS data. Model 3 with older age, higher BMI, earlier positive testing time, and a history of chronic heart failure, atrial fibrillation, and diabetes showed a good fit to the data (Hosmer and Lemeshow Goodness-of-Fit p values = 0.494, AIC = 584.3). Model 4 with WGS data has the best ROC curves, indicating that it discriminates severe COVID-19 better than the other models by improving the risk discrimination of severe COVID-19 from ~68.1% to 71.5% (Hosmer and Lemeshow Goodness-of-Fit p values = 0.715, AIC = 345.7). Based on clinical plausibility, and statistical tests results, we selected Model 3 with EHR data and Model 4 with EHR plus WGS data as the final models. (Table 3).

## Discussion

In this study, we found that among people hospitalized with COVID-19, the model we developed could predict COVID-19 severity based on older age, higher BMI, earlier test positive period (March-June 2020), and history of chronic heart failure, atrial fibrillation, or diabetes. The finding in bivariate analysis that use of dexamethasone, remdesivir, BiPaP, hydroxychloroquine, and tocilizumab were more common among case patients than controls reflects the fact that these therapies were preferentially given to patients with more severe COVID-19. The analysis that included patients with WGS data did not appreciably change the results of the above risk factor analysis. Several SARS-CoV-2 genomic characteristics were independently associated with severe COVID-19, including lineages B.1 and B.1.2, as well as clade 20A. Several mutations were also associated with worse outcomes, however none of those mutations were associated with severe COVID-19 in our final adjusted model.

Increased risk of severe COVID-19 among older patients in this study corroborates earlier findings [5, 17]. We also found that higher BMI was associated with severe COVID-19, which is in agreement with previous studies [6]. Consistent with the literature, our study found no association between race and severe COVID-19 among hospitalized patients [5, 6, 18]. We found that underlying medical conditions increased the risk of severe COVID-19, including chronic heart failure, atrial fibrillation, diabetes, and obesity (when adjusting for age). Additionally, each 1 unit increase in CCI increased the risk of severe COVID-19 by about 14% after adjustment for age. These findings are in agreement with recent studies concluding that a higher number of underlying medical conditions increased the risk of ICU admission or mortality [5]. Unlike some other studies, we did not detect an association between male gender and severe COVID-19 [5, 6, 19, 20]. A possible explanation for this discrepancy might be that male patients in our cohort had a lower BMI than female patients (mean = 30 (SD = 8.0) vs. mean = 34 (SD = 15.1)), which might have balanced out their risk of severe COVID-19. Another important finding was that earlier test positive period (March-June 2020) was strongly associated with increased risk of severe COVID-19, with the highest risk occurring earliest during the pandemic (March-June 2020). The lowest risk (75% less likely to have severe COVID-19 compared to earlier time) was observed during November 2020-Feburary 2021, which likely reflects improved care of COVID-19 patients over time. By the end of February 2021, 14.9% of the population in Pennsylvania had at least 1 dose of COVID-19 vaccination and most vaccinated individuals were health care workers, therefore the lower risk of severe COVID-19 outcome is less likely due to COVID-19 vaccination [21].

We found no significant association between SARS-CoV-2 mutations and COVID-19 severity in our adjusted model. A limited number of prior studies have examined genomic factors associated with COVID-19 severity. A case-control study by Dite et al., using UK biobank data evaluated a panel of human genome markers, in addition to clinical and demographic variables, to develop a model to predict risk of severe COVID-19. They reported that age, gender, comorbidities, and SNP score discriminated severe COVID-19 from non-severe better than clinical factors or SNP score alone [12]. This finding broadly supports the work of those previous studies linking certain genomic factors with severe COVID-19 [12]. Although it is possible that these findings have biologic significance, it is also possible that these are chance findings in a study that examined many factors associated with severe infection.

This study has several limitations. First, the included patients were all treated within a single health system; the risk factors associated with severe COVID-19 might differ in regions with patient populations with different characteristics. Second, our analysis was restricted to only hospitalized patients. All patients were treated by the evolving standards of care, and our findings do not imply anything about the natural course of the disease of whether other variants are more or less severe in the absence of hospital care. Third, our study was performed before the emergence of the SARS-CoV-2 Delta and Omicron variants in our region and nationally and, therefore, was unable to address whether these variants were associated with more severe disease outcomes. Fourth, the sequences were limited to those patients who presented with higher viral loads, as we could only sequence those with Ct < 33.

In conclusion, we found that older age, higher BMI, early test positive period, history of chronic heart failure, atrial fibrillation, or diabetes; and infection with clade 20A SARS-CoV-2 strains were significantly associated with severe COVID-19 among hospitalized patients. Our findings could be used to identify patients with higher risk of severe COVID-19 to prioritize patients for prophylaxis, early therapy, and efforts to improve SARS-CoV-2 immunization rates.

## Supporting information

S1 Fig Distribution of SARS-CoV-2 lineages over time (March 2020 to May 2021), by case-control status, UPMC. (TIFF)
