# Supplementary figures and images for: Predictive model for severe COVID-19 using SARS-CoV-2 whole-genome sequencing and electronic health record data, March 2020-May 2021
Source: PLoS One. 2022 Jul 12;17(7):e0271381. doi: 10.1371/journal.pone.0271381 (PMC9275682; doi:10.1371/journal.pone.0271381)

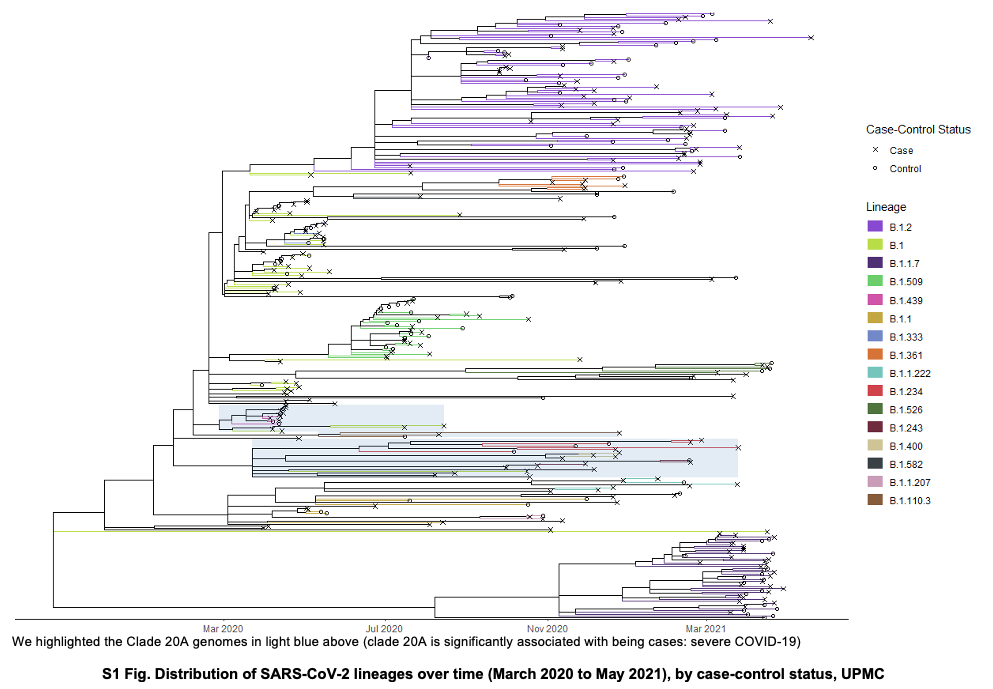

Supplement: S1 Fig — (TIFF) [file pone.0271381.s001.tiff]
